# Spontaneous rupture and hemorrhage of a giant simple hepatic cyst in a 94-year-old woman: a case report

**DOI:** 10.3389/fsurg.2026.1940004

**Published:** 2026-09-18

**Authors:** Jingfeng Qiu, Changzhi Chen, Lin Li

**Affiliations:** Department of Hepatobiliary and Pancreatic Surgery, The Third Affiliated Hospital of Guangxi Medical University, Nanning, China

**Keywords:** elderly, hemorrhage, rupture, simple hepatic cyst, spontaneous

## Abstract

**Background:**

Spontaneous rupture and hemorrhage of a simple hepatic cyst is a rare but potentially life-threatening complication, particularly in elderly patients. Because its clinical and imaging findings may mimic other complex cystic liver lesions, timely diagnosis and appropriate intervention are essential.

**Case presentation:**

A 94-year-old woman presented with chronic upper abdominal pain that suddenly worsened. Computed tomography revealed a giant complex cystic lesion measuring approximately 20 cm in the left hepatic lobe, with internal high-density areas and thin septations. The differential diagnosis included a complicated hepatic cyst with intracystic hemorrhage and mucinous cystic neoplasm of the liver (MCN-L). The patient initially remained clinically stable; however, she subsequently developed worsening abdominal pain, chest tightness, shortness of breath, hypotension, tachycardia, and a decrease in hemoglobin from 85 g/L to 76 g/L. Contrast-enhanced computed tomography demonstrated massive intraperitoneal fluid consistent with hemoperitoneum. Emergency laparoscopic exploration revealed approximately 1,000 mL of intraperitoneal blood and a ruptured giant left hepatic cyst filled with blood clots. Laparoscopic left hepatectomy was performed. Postoperative pathology confirmed a simple hepatic cyst. The patient recovered uneventfully and was discharged on postoperative day 9.

**Conclusion:**

Spontaneous rupture with hemorrhage should be considered in elderly patients with giant hepatic cysts who develop acute abdominal pain and unexplained hemoglobin decline. Emergency laparoscopic surgery may provide definitive treatment, control bleeding, and allow pathological confirmation.

## Introduction

Simple hepatic cysts are common benign liver lesions and are usually asymptomatic. Most are detected incidentally during abdominal imaging and require no treatment (1, 2). However, large cysts may cause symptoms due to compression of adjacent structures, including the biliary tract, gastrointestinal tract, vessels, or diaphragm. Less common complications include intracystic hemorrhage, infection, rupture, and, rarely, hemoperitoneum (3, 4). Spontaneous rupture and hemorrhage of a simple hepatic cyst is rare but may be life-threatening. The proposed mechanism involves intracystic hemorrhage, rapid elevation of intracystic pressure, ischemic thinning of the cyst wall, and eventual rupture into the peritoneal cavity. Giant cyst size is considered an important predisposing factor. Because the clinical presentation may be nonspecific and imaging findings may mimic cystic neoplasms, timely recognition remains challenging, especially in elderly patients with comorbidities.

Here, we report a 94-year-old woman with spontaneous rupture and hemorrhage of a giant simple hepatic cyst, successfully treated by emergency laparoscopic left hepatectomy. This case highlights the importance of considering rupture in elderly patients with giant hepatic cysts, acute abdominal pain, and unexplained hemoglobin decline.

## Case report

A 94-year-old woman presented to our hospital on May 6, 2025, with upper abdominal pain for more than one year, which had suddenly worsened six hours before admission. Her medical history included chronic kidney disease and neck surgeries approximately 50 years earlier. No history of abdominal surgery was reported, and she had not been taking any anticoagulant or antiplatelet medications. On admission, her abdominal pain had partially improved. Physical examination revealed mild upper abdominal tenderness without rebound tenderness. A palpable mass was noted below the xiphoid process and in the right hypochondrium. Laboratory tests on admission showed a hemoglobin level of 85 g/L. Because no recent pre-event hemoglobin measurement was available, the chronicity of the anemia could not be definitively established. Platelet count was 132 × 10⁹/L. Coagulation profiling demonstrated prolonged prothrombin time (15.4 s), elevated international normalized ratio (INR, 1.22), elevated fibrinogen (5.46 g/L), and markedly increased D-dimer (1.91 mg/L), while activated partial thromboplastin time remained within normal limits. Serum creatinine was 122 μmol/L, lipase level was 170.6 U/L, and liver enzyme levels were normal. Initial abdominal computed tomography revealed a giant complex cystic lesion in the left hepatic lobe, measuring approximately 20 cm in diameter. Patchy high-density areas and thin linear septations were observed within the lesion (Figure 1). Based on the imaging findings, a diagnosis of a complex hepatic cyst was considered (5, 6). Because the patient was initially hemodynamically stable, close observation was chosen after discussion with the patient and her family.

**Figure 1:**
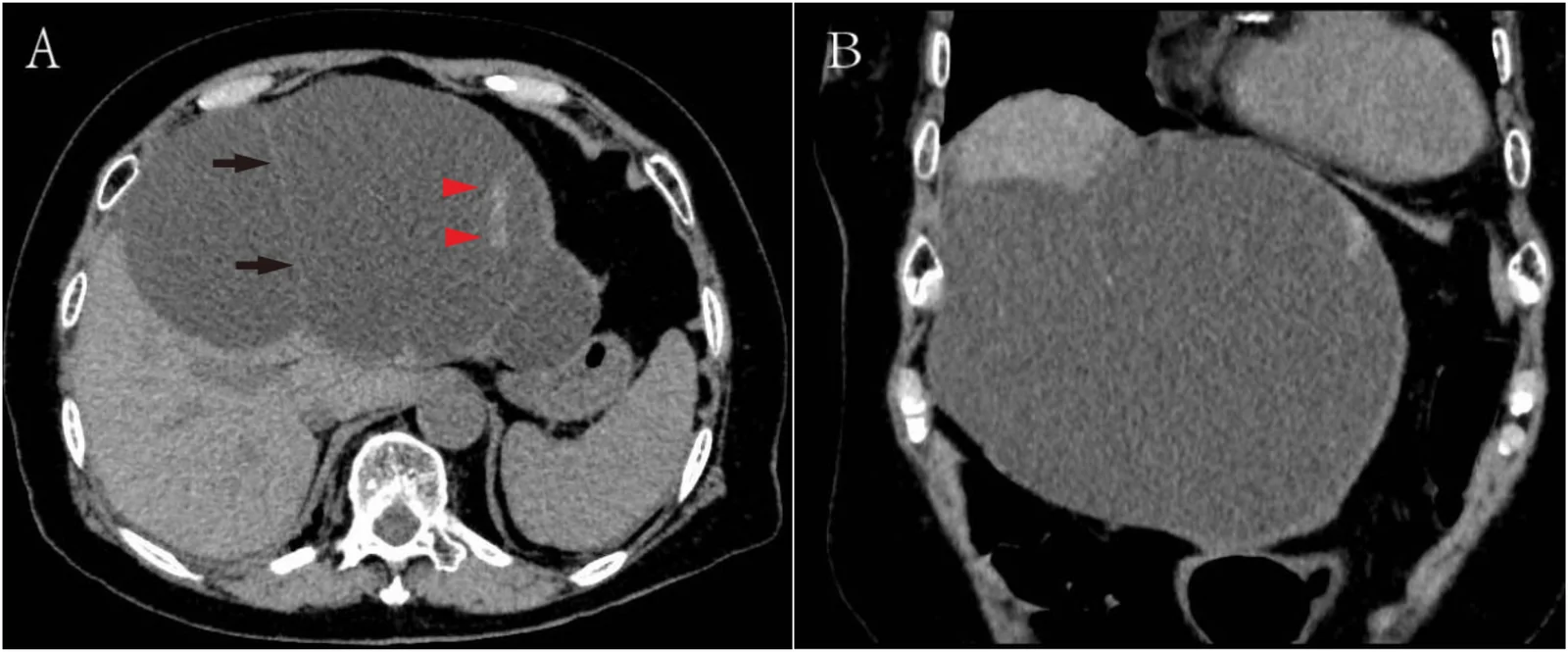
Non-contrast computed tomography showing a 20-cm complex cystic lesion in the left hepatic lobe. Patchy high-density areas and thin linear septations are visible within the cyst. **(A)** Axial view. **(B)** Coronal view.

On May 7, 2025, the patient suddenly experienced chest tightness, shortness of breath, and abdominal pain. Her blood pressure was 91/55 mmHg, heart rate was 106 bpm, and laboratory tests indicated a drop in hemoglobin (76 g/L). Immediate resuscitative measures consisted of intravenous fluid administration for hemodynamic support; no allogeneic blood transfusion was administered, though blood products were cross-matched and reserved for potential intraoperative use. Vasopressor agents were not required during this acute episode. Enhanced CT demonstrated a large amount of free intraperitoneal fluid (blood), indicating a recent rupture with hemoperitoneum. No nodular mural thickening, nodular septal enhancement, intralesional solid component or peripheral rim-enhancement was identified within the cyst. No active contrast extravasation was seen at the time of imaging (Figure 2). Emergency laparoscopic exploration was undertaken. Upon abdominal entry, approximately 1,000 mL of hemoperitoneum and a giant ruptured left-hepatic-lobe cyst containing abundant intralesional clots were encountered. No discrete active arterial bleeding site was identified intraoperatively, consistent with the pre-operative CT finding of absent contrast extravasation. The cyst predominantly involved hepatic segments S2-S4. The cyst arose from the hepatic parenchyma of segments II-IV; most of the lesion consisted of thin-walled cystic tissue, with only a thin rim of compressed adjacent hepatic parenchyma, and dense adhesions between the superior cyst wall and the diaphragm. Given the large cyst dimension, extensive parenchymal adhesion to the diaphragm, and inability to definitively rule out underlying cystic neoplasm, laparoscopic left hepatectomy with complete resection of the cyst wall was selected over alternative strategies including laparoscopic cyst fenestration/deroofing, isolated surgical hemostasis, or angiographic embolization. Fenestration alone was deemed insufficient given the high risk of persistent hemorrhage and recurrent cyst-related complications, whereas embolization was less favorable in the absence of identifiable arterial bleeding focus. Hepatic parenchymal transection was performed using an ultrasonic surgical device; parenchymal and cut-surface hemostasis was achieved with a combination of bipolar electrocoagulation, titanium clips, and topical hemostatic material applied onto the raw liver surface. The total operative duration was 130 min, with estimated intraoperative blood loss of 30 mL (distinct from the pre-existing 1,000 mL hemoperitoneum); no peri-operative allogeneic blood or blood-product transfusion was administered. No intraoperative complications were encountered. Post-operatively, hemostasis was secured with adequate abdominal drainage, and no liver-related surgical complications were observed during the in-hospital course. Postoperative pathological examination showed a fibrous cyst wall lined by flat-to-cuboidal bland biliary-type epithelium, without ovarian-type stroma, mucinous proliferation or cellular atypia. Immunohistochemical staining was not performed in this case. Morphological features alone supported the diagnosis of a simple hepatic cyst (Figure 3). Pre-operatively, comprehensive peri-operative risk assessment was performed considering the patient's advanced age (94-year-old) and pre-existing chronic kidney disease. Multidisciplinary geriatric-anaesthetic optimization was conducted prior to emergency surgery. After the operation, the patient was admitted for close postoperative monitoring, including haemodynamic surveillance, serial haemoglobin measurement, abdominal drain output recording, and repeated renal-function laboratory tests. Her preoperative serum creatinine was 122 μmol/L; post-operative serum creatinine was 127 µmol/L, without progressive deterioration of renal function. Urine output remained satisfactory throughout hospitalization. No new-onset renal injury or requirement for renal-replacement therapy occurred during the in-hospital stay. The patient was discharged on postoperative day 9 following satisfactory in-hospital recovery. At the 6-month follow-up, she remained asymptomatic, with no evidence of recurrence (Table 1).

**Figure 2:**
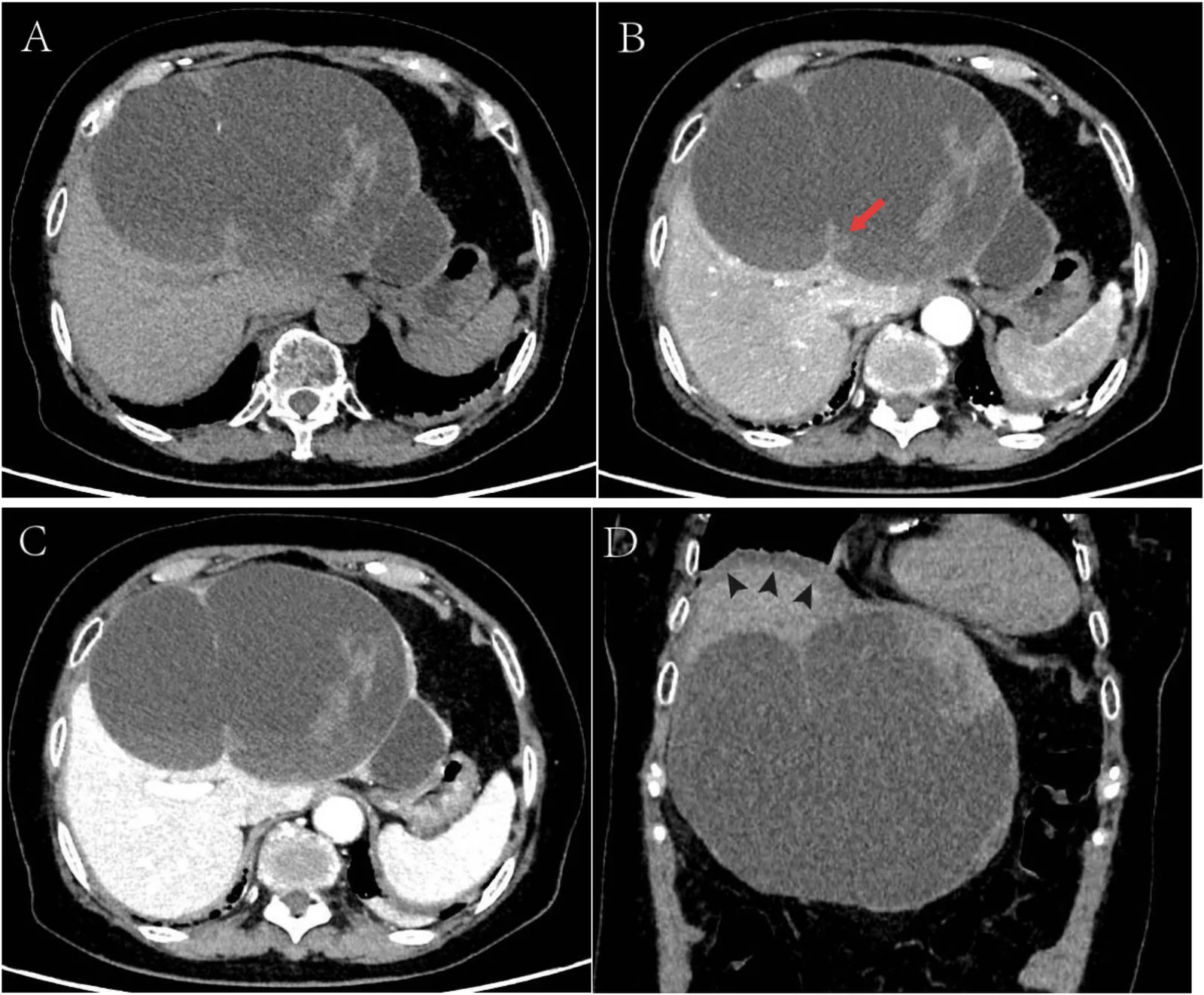
Dynamic contrast-enhanced computed tomography findings. **(A)** Non-contrast phase. **(B)** Arterial phase; the red arrow indicate the area of intra-abdominal hemorrhage, where large volumes of blood clots accumulated intraoperatively. This clot-rich region does not represent a direct visualization of the cyst rupture site. No discrete cyst-wall defect was identifiable on preoperative CT across all contrast phases. Neither septal enhancement nor cyst-wall enhancement was detected. **(C)** Delayed phase. **(D)** Coronal non-contrast image showing high-density perihepatic fluid consistent with hemoperitoneum.

**Figure 3:**
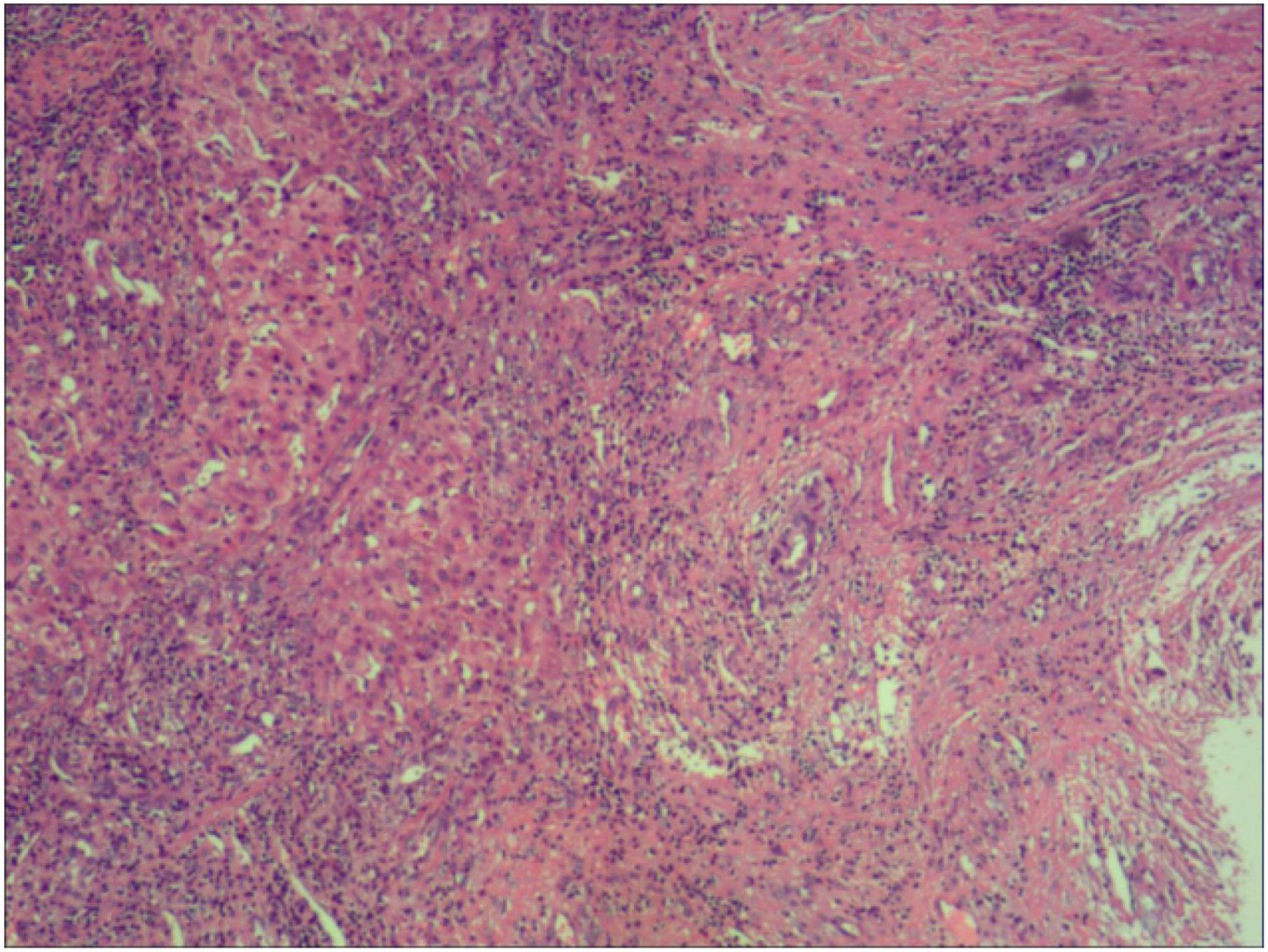
Histopathological examination showing a cyst wall lined by bland biliary-type cuboidal to low columnar epithelial cells, with underlying fibrous tissue hyperplasia and mild chronic inflammatory infiltration, consistent with a simple hepatic cyst. Hematoxylin and eosin staining, original magnification  × 100.

**Table 1:** Clinical timeline of the patient.

Date	Clinical events	Key investigations	Management
May 6 (Admission)	Elderly female, upper abdominal pain worsened 6 h prior; pain partially improved. Haemodynamically stable. Mild upper abdominal tenderness, no rebound; palpable mass.	Hb 85 g/L, platelets 132 × 10⁹/L, PT 15.4 s, INR 1.22, D-dimer 1.91 mg/L, creatinine 122 μmol/L. CT: giant complex cystic lesion (∼20 cm) in left hepatic lobe (Figure 1).	Close clinical observation chosen after discussion.
May 7 (acute deterioration)	Sudden chest tightness, dyspnoea, abdominal pain. BP 91/55 mmHg, HR 106 bpm.	Hb dropped to 76 g/L. Enhanced CT: massive haemoperitoneum consistent with cyst rupture; no active extravasation (Figure 2).	IV fluid resuscitation; blood products cross-matched. Emergency laparoscopic surgery performed.
May 7 (intra-operative)	∼1,000 mL hemoperitoneum; giant ruptured left-lobe cyst (S2-S4) with intralesional clots; no active arterial bleeding.	Intra-operative inspection.	Laparoscopic left hepatectomy. Blood loss 30 mL; no transfusion.
Post-operative & follow-up	Satisfactory recovery; no liver-related complications.	Pathology: simple hepatic cyst (Figure 3).	Discharged on postoperative day 9; asymptomatic at 6-month follow-up.

## Discussion

Spontaneous rupture and hemorrhage of a simple hepatic cyst is rare, but it may become life-threatening when hemoperitoneum develops (7–10). The present case is notable for three reasons. First, the patient was 94 years old, making emergency surgical decision-making particularly challenging. Second, the hepatic cyst was giant, measuring approximately 20 cm in diameter. Third, despite advanced age and acute intra-abdominal hemorrhage, the patient was successfully treated by emergency laparoscopic left hepatectomy and recovered without major postoperative complications.

The initial diagnosis was challenging. Simple hepatic cysts are common benign liver lesions and are usually asymptomatic; however, large cysts may cause symptoms, and intracystic hemorrhage may alter their imaging appearance (1, 2). In this case, computed tomography showed a giant cystic lesion with patchy high-density areas and thin septations, raising concern for a complicated hepatic cyst with hemorrhage, MCN-L, or other cystic neoplasms. These entities may overlap radiologically, especially when hemorrhage or proteinaceous fluid accumulates within the cyst (5, 6, 11, 12). Nevertheless, preoperative imaging lacked thickened or nodular septa, mural nodules, and cyst-wall or septal enhancement, imaging features that may raise suspicion for MCN-L according to current EASL guidance (13). Histopathological examination of the resected specimen demonstrated a fibrous cyst wall lined by bland biliary-type epithelium. No mucinous epithelial proliferation, cellular atypia, or ovarian-type stroma, a key histopathological feature of MCN-L, was identified (14). These findings supported the diagnosis of a complicated simple hepatic cyst and argued against MCN-L and other cystic neoplasms. These microscopic findings ruled out MCN-L and other cystic neoplastic entities and established the final diagnosis of a complicated simple hepatic cyst. Therefore, in elderly patients with giant hepatic cysts and complex imaging features, dynamic clinical observation and repeated imaging are important, particularly when symptoms worsen or hemoglobin decreases. We further considered and excluded other differential diagnoses based on clinical, laboratory and imaging data. MCN-L was considered less likely because contrast-enhanced CT showed no nodular septal thickening, mural nodules, or cyst-wall or septal enhancement. No history of echinococcal exposure, no peripheral calcification of cyst wall, no daughter cysts on CT made echinococcal cyst unlikely. Liver abscess was ruled out by the absence of fever, normal white-blood-cell count and lack of characteristic rim-enhancement and perilesional inflammatory changes on CT. The complex imaging appearance was fully attributable to intracystic hemorrhage rather than other primary cystic neoplasms.

The mechanism of rupture in this case was likely related to intracystic hemorrhage and increased intracystic pressure. A giant cyst may place persistent tension on the cyst wall, and hemorrhage into the cyst can further increase internal pressure. This process may lead to ischemic thinning, necrosis, and eventual rupture of the cyst wall, allowing hemorrhagic contents to enter the peritoneal cavity (4, 7–10). The patient's sudden worsening abdominal pain, chest tightness, tachycardia, blood pressure reduction, hemoglobin decline, and CT evidence of hemoperitoneum were consistent with this process. No definite precipitating factor was identified.

Management of spontaneously ruptured simple hepatic cysts remains heterogeneous, and treatment strategies are largely derived from retrospective case series and recent observational reports, given the absence of randomized controlled trials (15, 16). Four main therapeutic modalities are currently available: conservative management, percutaneous drainage with or without sclerotherapy, laparoscopic fenestration, and hepatic resection, each with distinct indications, benefits, limitations and recurrence risks. Conservative management, consisting of fluid resuscitation, pain control and close haemodynamic monitoring, is reserved for haemodynamically stable patients with limited intra-peritoneal bleeding and no evidence of ongoing haemorrhage. Its main advantage is avoidance of procedural-related risks; however, it carries risks of re-bleeding, persistent abdominal symptoms and delayed diagnosis when cystic neoplasm cannot be excluded. Percutaneous drainage combined with sclerotherapy is minimally invasive and suitable for high-risk patients unfit for general anaesthesia; nevertheless, in the setting of acute rupture with haemoperitoneum, it cannot evacuate intra-abdominal free blood, cannot achieve complete cyst-wall removal, and is associated with comparatively high recurrence rates. Laparoscopic fenestration (deroofing) is the standard procedure for uncomplicated symptomatic simple hepatic cysts; it is less invasive than formal liver resection and yields acceptable outcomes for selected patients. However, fenestration leaves residual cyst wall *in situ*, which may predispose to persistent or recurrent haemorrhage in acutely ruptured, giant, haemorrhage-filled cysts. Moreover, fenestration cannot provide full-specimen histopathological evaluation to definitively rule out underlying cystic neoplasms. Hepatic resection achieves complete removal of the cyst-bearing hepatic parenchyma, allows thorough evacuation of haemoperitoneum, provides definitive histopathological diagnosis, and offers the lowest long-term recurrence risk; the major drawback is higher technical demand and inherent risks related to liver resection, which should be weighed against patient physiological status (15, 16).

Against this background, laparoscopic left hepatectomy rather than fenestration or interventional therapy was chosen for our patient for several explicit reasons. First, the cyst was huge (20 cm) with substantial intra-peritoneal haemorrhage and clinical deterioration, and preoperative imaging could not fully exclude cystic neoplasia. Complete resection enabled one-stage evacuation of haemoperitoneum, source control, total cyst-wall excision and definitive pathological diagnosis simultaneously. Second, laparoscopic fenestration was considered suboptimal: residual cyst wall after fenestration would leave behind haemorrhage-prone tissue, raising concerns for persistent or recurrent bleeding. Third, percutaneous drainage or transcatheter embolization was not appropriate in this clinical scenario: there was no identifiable active arterial bleeding focus on contrast-enhanced CT, and interventional approaches could not resolve free intra-abdominal haemoperitoneum nor obtain complete tissue for histology. Despite the patient's advanced age and baseline chronic kidney disease, her physiological reserve permitted emergency laparoscopic liver resection. Compared with simple drainage or fenestration, definitive surgical resection may reduce recurrence risk and enable pathological confirmation, particularly when cystic neoplasm cannot be excluded preoperatively (15, 16).

The successful outcome in this case suggests that advanced age alone should not constitute an absolute contraindication to emergency laparoscopic surgery in carefully selected individuals. For very elderly patients, treatment decisions should be based on physiological reserve, comorbidities, hemodynamic status, cyst anatomical features, and concern about underlying neoplasia, alongside local surgical expertise. Although prior literature has reported potential benefits of laparoscopy relative to open surgery (17), conclusions regarding superior trauma reduction or faster recovery cannot be drawn from this single-case observation. In our patient, comprehensive peri-operative optimization and timely intervention collectively contributed to the uneventful postoperative course.

This case provides several clinical lessons. First, rupture should be considered when an elderly patient with a giant hepatic cyst develops acute abdominal pain, hemodynamic changes, or an unexplained decrease in hemoglobin. Second, complex imaging features in a hepatic cyst do not always indicate neoplasia; intracystic hemorrhage can mimic cystic tumors (5, 6, 8, 12). Third, emergency laparoscopic resection can serve as a definitive treatment option for select patients with ruptured giant hepatic cysts, as illustrated in this individual case; it is not universally preferred over fenestration, embolization or alternative management strategies. Treatment selection must balance hemodynamic stability, evidence of ongoing bleeding, cyst location, and suspicion for neoplastic transformation. Finally, pathological confirmation remains important when preoperative imaging cannot confidently exclude cystic neoplasm.

This report has several limitations. First, it describes a single case, and the findings cannot be generalized to all elderly patients with ruptured hepatic cysts. Second, the exact trigger for cyst rupture remained unclear. Further accumulation of similar cases is needed to clarify risk factors, optimal timing of intervention, and the best treatment strategy for ruptured giant hepatic cysts in elderly patients.

## Conclusions

Spontaneous rupture and hemorrhage is a rare but serious complication of giant simple hepatic cysts. In elderly patients with giant hepatic cysts, acute abdominal pain and unexplained hemoglobin decline should raise suspicion of rupture. This case suggests that emergency laparoscopic surgery can be a feasible and definitive treatment option in carefully selected very elderly patients, allowing bleeding control, lesion removal, and pathological confirmation.

## Data Availability

The original contributions presented in the study are included in the article. Further inquiries can be directed to the corresponding author.
